# Overview of vehicle submersion in natural waters: challenges, associated risk factors, escape strategies, and general recommendations

**Published:** 2024-10

**Authors:** Faride Sadat Jalali, Zahra Zare, Mohadeseh Ghanbari-Jahromi

**Affiliations:** ^*a*^ Health Human Resources Research Center, Shiraz University of Medical Sciences, Shiraz, Iran.; ^*b*^ Student Research Committee, Shiraz University of Medical Sciences, Shiraz, Iran.; ^*c*^ Research Center for Social Health Determinants, Jahrom University of Medical Sciences, Jahrom, Iran.

## Abstract

**Background:**

Accidents leading to drowning are uncommon, and limited information is available on drownings associated with road traffic accidents. Therefore, this study aimed to investigate the challenges, risk factors, escape strategies, and general recommendations related to vehicle immersion in natural waters.

**Methods:**

This systematic review was conducted following the PRISMA-SCR (Preferred Reporting Items for Systematic Reviews and Meta-Analyses extension for Scoping Reviews) guidelines. A comprehensive literature search using relevant keywords was performed in the PubMed, Scopus, Web of Science, ProQuest, SID, and Magiran databases up to July 2024. Articles were selected based on inclusion criteria, and after removing duplicates and screening titles, abstracts, and full texts, 22 articles were analyzed.

**Results:**

The findings indicated that the challenges of vehicle drowning include difficulties in identifying the cause of death, ineffective action protocols, and delays in the immediate dispatch of rescue efforts for drowning vehicles. Risk factors associated with vehicle immersion include distraction, driver drowsiness, high speed, accidents, parking on riverbeds and seabeds, and driving on ice or roads near water. Escape strategies involve the "POGO" method (fasten seat belt, open or break windows, and exit), the "SWOC" method (fasten seat belt, open or break windows, exit the vehicle, with priority given to children), and general safety measures such as fastening seat belts, rolling down windows, unlocking car doors, and climbing out. Additionally, general recommendations for drivers include keeping a small metal hammer in the car for breaking windows, avoiding travel during storms, and refraining from driving on flooded roads.

**Conclusions:**

The results indicate that public awareness of vehicle drowning incidents is low. Enhancing public education, improving road design, adding proper markings and lighting, installing guardrails, providing flood warnings, promoting cautious driving practices, and updating current protocols could help reduce such accidents.

**Keywords:**

Drowning, Submersion, Vehicles, Automobile, Car

